# Annotations

**Published:** 1911-02-04

**Authors:** 


### Annotations.

### The Social Value of the Premature.

Guibert, in a Thèse de Lyon, discusses the social value of children born as the result of labour prematurely induced in cases of contracted pelvis. Some authorities have maintained that such premature infants live only to become imbeciles, idiots, or epileptics, but the observations carried out by the author in the clinic of Professor Fabre do not support these assertions. Of the forty-four children, varying in age from two to thirteen years, examined by Guibert, only four have died, and of the rest one only is of defective physical and one of defective mental development. It would thus seem established that the premature can eventually attain a physical and mental standard quite on a plane with that of normal children. The author, however, insists that to obtain the best results delivery should be delayed as long as is compatible with safety to the mother. Delivery can be provoked after the thirty-third week of gestation, at a time when the child should weigh about 4¾ lbs. Induced labour is justifiable in cases where the generally contracted pelvis has a useful diameter of 9 centimetres (3½ inches), in a generally contracted and flattened pelvis of 8 centimetres (3.12 inches), and in a flattened pelvis of 7 centimetres (2.73 inches) diameter. It must be borne in mind that the premature infant above all others is in want of the greatest care and breast-feeding, and that all the usual rules must be applied in its case, and in particular that which limits its feeding to once in every three hours.

### The Doctor as Patient.

"Physician heal thyself" is a phrase which almost irresistibly rises to the lips of some platitudinous person when the doctor himself becomes a patient. It is generally spoken in a tone of gentle raillery at the helplessness and inconsistency of the medical mind when the medical body is stretched upon a bed of sickness. It has much in common with the cheap sneer so often expressed by laymen when they find the "medical oracle" himself flouting some rule of health which he has laid down under certain circumstances for the guidance of others. When the doctor is ill it goes without saying that his patients are sorry for him in his affliction; but at the same time some of them will hint that it was just a little inconsiderate of him, if not a little incompetent as well, to leave his patients to the care of an outsider at such short notice. Illness is indeed a dreadful calamity for the practitioner, and it so often comes at a time of great mental and physical stress. It is small wonder, therefore, that he above all others—in spite of his special knowledge, or because of it—should become particularly demoralised between the time when he suspects in himself the first vague symptoms of disease and the time when he knows that he is really ill and must give up work. He is too near his own case to judge of it clearly and calmly and deliberately. As likely as not he begins by tinkering half-heartedly at his system with a dozen remedies in quick succession, or else, with the disease working upon him, he runs hither and thither like a scared animal from one quarter of the pharmacopœia to another, until at last his poisoned wits dimly perceive that the matter is beyond him and other help is needed. Is it really surprising that doctors sometimes make bad patients, especially in the early stages of disease?

### A Medical Composer.

From time to time members of the medical profession achieve fame and fortune in other fields. Only too often the result is that we lose from our number those who would doubtless have attained distinction, even eminence, in the profession, if the more alluring prospects of early success had not induced them to abandon it for the pursuit of literature, art, science, or what not. Sir Charles Wyndham, surely, must have done well in his old profession had not the boards of the stage tempted him away. Sir A. Conan Doyle's solid common sense and vigorous personality, too, must have ensured his ultimate advancement as a practitioner. Sir R. B. Finlay's talents, unquestionably, would have carried him far in his medical career had he not espoused the law. The list could be multiplied indefinitely; suffice it for the moment to note that the prize of £100 offered by the *Evening News* for a new waltz by a British composer has been won by a young London practitioner, Mr. Ivan Maclean, M.B., B.S. This gentleman is an old St. Thomas's man, lately on the resident staff of the West London Hospital. Without offence, the hope may be expressed that Dr. Maclean may find it possible to allow musical composition to remain a pleasant (and apparently profitable) relaxation rather than to desert a profession of which he may quite possibly become a distinguished ornament.

### Turkish Court Physicians.

A case is about to come on for trial at Constantinople which shows that Abdul Hamid pushed somewhat to extremes his taste for experiments *in anima vili*. Some years back, at the height of his power, when one of his best-loved daughters fell ill with appendicitis, the Sultan declined to give permission for an operation to be performed upon the sultana before being shown exactly in what it consisted and that it was as simple a matter as had been made out. The court practitioners suddenly bethought themselves of a woman, an Ottoman subject, who was about to be discharged convalescent from the Italian hospital after suffering from some liver complaint. She was seized, taken to the Hamidia hospital, and, in the presence of the Sultan, her appendix was removed. The operation was a complete success, the patient, or rather the victim, recovering quickly, and the Sultan, in consequence, allowed his daughter to be treated in similar fashion. To-day Abdul Hamid is banished, tongues are untied, and his victim is bringing an action against the court physicians who risked her life by their experiment. The decision of the tribunal will be of interest.

